# What motivates people to commence a graduate entry nursing programme: a mixed method scoping review

**DOI:** 10.1186/s12912-021-00564-9

**Published:** 2021-03-20

**Authors:** Rachel Macdiarmid, Rosemary Turner, Rhona Winnington, Patricia McClunie-Trust, Andrea Donaldson, Kay Shannon, Eamon Merrick, Virginia Jones, Rebecca Jarden

**Affiliations:** 1 Department of Nursing, Faculty of Health and Environmental Science, Auckland University of Technology, 90 Akoranga Drive, Northcote, Auckland, 0627 New Zealand; 2 Department of Nursing, Faculty of Medicine, Dentistry and Health Sciences, 161 Barry Street, The University of Melbourne, Victoria, 3010 Australia; 3 Centre for Health and Social Practice, Waikato Institute of Technology, Tristram Street, Hamilton, 3240 New Zealand; 4 Centre for Postgraduate Nursing, University of Otago, 72 Oxford Terrace, Christchurch, 8052 New Zealand

**Keywords:** Nursing, Education, Graduate, Students, Motivation, Nurse education, Nursing student

## Abstract

**Background:**

The global deficit of nurses demands urgent attention in the recruitment and education of this future workforce. Graduate entry nursing (GEN) programmes are one option for people with undergraduate degrees who are seeking nursing education. Determining the key motivations for enrolling in these programmes will support the development of new initiatives in the education sector to both recruit and retain this future workforce and inform future primary research. This scoping review aims to comprehensively describe what motivates graduates to enrol in GEN programmes.

**Methods:**

Peer reviewed studies of quantitative, qualitative and mixed-method research investigating motivations to commence a graduate entry nursing programme were included, following a pre-determined protocol. Electronic databases searched included Cumulative Index to Nursing and Allied Health Literature (CINAHL), Emcare, ERIC, Medline and Scopus. Screening, data extraction and analysis was initially in duplicate and independent, then consensus reached. Qualitative and quantitative data was analysed and reported separately then combined thematically as a narrative synthesis in a convergent segregated approach. Reporting followed preferred reporting guidelines for scoping reviews.

**Results:**

Of the 491 studies retrieved in July 2020, across the five databases and reference list search, six met the inclusion criteria. Four were qualitative studies, one mixed-methods, and one quantitative, respectively from Australia, USA, and New Zealand. Four themes of motivation were identified: 1) finding meaning and purpose through altruism and caring; 2) seeking a satisfying career, 3) looking for a change in direction and, 4) reduced financial burden due to course length and provision of scholarships.

**Conclusions:**

There is a paucity of studies specifically seeking to investigate student motivations for enrolling in a GEN programme and only limited studies giving insights into motivators for enrolling in a GEN programme, therefore this scoping review contributes new understandings on the reason’s students choose GEN programmes. These are both altruistic and practical and include personal desires to help others, the need to pursue a satisfying and meaningful career and the shorter period out of the workforce offered by an accelerated programme of study.

**Supplementary Information:**

The online version contains supplementary material available at 10.1186/s12912-021-00564-9.

## Background

Currently, the global shortage of nurses is reported as a 5.9 million deficit [1]. The projected numbers of nursing graduates need to increase by an average of 8% each year to meet healthcare demand [1]. To meet this shortfall the ICN has called for support for funding the education and employment of a greater number of nurses [1]. One potential strategy to achieve this is the establishment of Graduate Entry Nursing (GEN) programmes.

Graduate entry nursing programmes are well established in Australia, the United Kingdom (UK) and the United States of America (USA) [2–4]. These programmes are for graduates who wish to become Registered Nurses and obtain a professional post-graduate qualification. Graduate Entry programmes have been shown to produce well equipped, responsive and work ready nursing graduates [5, 6].

Since the commencement of GEN degrees there has been great interest in the students enrolled in these programmes. Areas of interest have focused on demographics of students and academic performance over 10 years [7]; and more recently gender-based differences in experiences and outcomes [8]. An integrative review of the literature has highlighted the developing knowledge of the demographics and characteristics of students entering a GEN programme [5]. What has not yet been established are students’ motivations for enrolling in GEN programmes.

It is timely to undertake this review due to the predicted workforce shortages [1]. The review exploring students’ motivations for enrolling in the GEN programmes will support the development of existing and future programmes to both suit and support learners needs, whilst meeting academic and regulatory body requirements. Identification of the key motivations for students enrolling in the GEN programmes will inform strategies for promotion and recruitment into programmes, programme development, and future research opportunities.

### Aim, objectives and review questions

The aim of this scoping review is to describe the evidence on the motivations of graduates who enrol in GEN programmes. The question of the review is: What are the motivations of graduates who enrol in GEN programmes?

## Methods

The scoping review adhered to an a priori protocol based on the recommendations of [9–11], the protocol was aligned with the Preferred Reporting Items for Systematic Reviews and Meta-Analysis Protocol (PRISMA-P) [12]. The current review integrates these frameworks with the Preferred Reporting Items for Systematic Reviews and Meta-Analysis extension for Scoping Reviews (PRISMA-ScR) checklist and explanation [13]. A three-stage approach to the search was guided by the recommendations of Aromataris & Munn [14].

The search strategy was tested in two electronic databases (CINAHL and Medline) to identify additional relevant keywords and index terms. The authors (RM and RJ) then conducted the search of keywords and index terms in all the included databases in July 2020. Two authors (RM and RJ) then reviewed the reference lists of included studies to identify any additional relevant studies.

All citation information was exported from databases to Endnote™ X8 (Clarivate Analytics, PA, USA) then to Covidence™ (Veritas Health Innovation, Melbourne, Australia) for identification of duplicate study results, screening and data extraction.

### Inclusion criteria

Types of participants included individuals seeking to enrol or who were enrolled in a graduate entry nursing programme. Key concepts included motivations, enablers and barriers. The contexts included anywhere applicants for a graduate entry nursing programme may be found such as tertiary education providers. The types of publications included peer-reviewed journal publications of quantitative, qualitative and mixed-methods primary research studies. No date nor language limiters were applied. Due to the large number of irrelevant studies from the veterinary science programmes, a ‘human/humans’ limiter was applied.

### Exclusion criteria

Studies were excluded where: 1) learners were entering undergraduate nursing degree programmes; 2) studies only presented academic or theoretical perceptions; 3) studies only presented learners experiences of a programme.

### Search strategy

The search of electronic databases included Cumulative Index to Nursing and Allied Health Literature (CINAHL), Emcare, ERIC, Medline and Scopus. Two reviewers independently screened all titles and abstracts, and full-text studies, for inclusion according to the pre-determined inclusion and exclusion criteria. The search strategy for Scopus is provided in Supplementary File 1. The search terms included the following keywords and associated index terms: nurs* AND “graduate entry”, “direct entry”, “G msn”, “MSN entry”, MNSc, “second degree”, “accelerated master*” AND motivat*, enabl*, barrier*, facilitat*, perception*, perceive*, aspiration*, attitude*.

### Study selection

To achieve consistency among reviewers, the first two included studies were independently screened (according to the inclusion and exclusion criteria) by both reviewers (RM and RJ) and the process and results discussed before continuing with the review. Thereafter, the two reviewers independently screened the titles and abstracts of the studies identified in the search followed by full text screening of those potentially meeting the inclusion criteria. Disagreements on study inclusion were resolved through discussion and consensus, consultation with a third reviewer was not required.

#### Quality assessment

The included studies were critically appraised independently by two reviewers (RM and RJ) to determine the risk of bias. The Joanna Briggs Institute critical appraisal tools were used by adding the questions from each relevant tool (qualitative, cross-sectional, and prevalence) to the Covidence™ custom risk of bias fields. Consensus was reached through discussion. Studies were not excluded based on the quality assessment.

### Data collection

Data from eligible studies were extracted independently by the two reviewers according to predetermined extraction fields entered into Covidence™. The fields included: 1) study characteristics such as date, location, population, research design, research objective/s and question/s, research measures; and 2) study findings such population demographics and outcomes. Consensus was reached through discussion.

#### Data analysis and reporting

Quality assessment findings are reported in tabular form with a narrative summary. Qualitative and quantitative data are reported independently, then analysed and reported in a narrative synthesis. Qualitative data were analysed thematically in a six-phase content-driven, inductive approach to the coding [15–17]. After reviewing and becoming familiar with the qualitative data, initial codes were identified (RM and RJ), drawing from authors themes and the explicit quotes by the participants. Two researchers (RM and RJ) categorised codes first independently, then compared and contrasted together. A third reviewer was not needed for consensus, however, all authors reviewed and discussed themes drawn from the codes. Qualitative data is reported thematically as a narrative summary then as part of the synthesis. Quantitative data is reported in tabular form with narrative summary then as part of the synthesis. Study screening and selection results are presented in a Preferred Reporting Items for Systematic Reviews and Meta-Analyses (PRISMA) flow diagram [12]. Extraction results are reported in tabular form with a narrative synthesis according participants, concept and context.

## Results

### Literature search

**Fig. 1:**
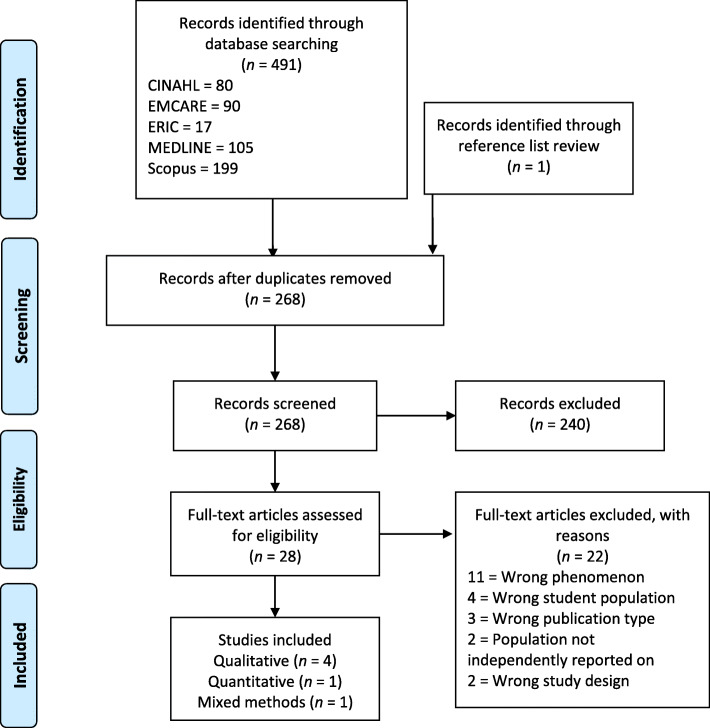
Flow chart of study selection

Database searches yielded 491 articles, with one further additional record from reference list searches. Following removal of duplicates, 268 publications remained, following title and abstract screening, 28 full text articles were assessed for eligibility. Six articles met the selection criteria and were included. A flow chart of the search screening process is illustrated in Fig. 1.

In the title and abstract screening, records were largely deemed irrelevant due to wrong population or phenomenon. During full text screening, 11 articles were excluded as they investigated the wrong phenomenon, four for wrong student population, and three were not research reports. Four studies were excluded because either the GEN students were not identifiable in the findings or GEN student motivations were not reported.

### Quality assessment

All quality assessments, including reviewer judgements, comments and consensus are reported in supplementary file 2. Of the four qualitative studies [18–21] the grounded theory study of Neill [20] was assessed as lowest risk of bias, meeting eight out of ten criteria. Participant voices were assessed as well represented across all four studies, however the position of the researcher within each study was not mentioned and only one study addressed the potential influence of the researcher on their findings [21]. Just one of the four qualitative studies reported philosophical perspectives or research methodology [20].

For the quantitative studies, the longitudinal study of DeWitty [22] provided insufficient data to determine adequacy of sample size, included no discussion of drop-outs, and did not address the small sample in some sub-groups in their analysis. All other criteria (6 out of 9) for their study were assessed as low risk of bias. McKenna and Vanderheide’s [23] cross-sectional survey was assessed as low risk of bias for six out of eight criteria. The two criteria assessed as high risk of bias were, firstly, they did not use validated measures (e.g., job satisfaction), and secondly, they did not follow conventions for reporting both means and standard deviations.

### Characteristics of included studies

The six included studies were conducted in New Zealand (*n* = 2), in the United States of America (*n* = 2), and Australia (*n* = 2), between the years 2011 and 2019, and were predominantly studies of qualitative descriptive design published in five nursing journals (see Table 1).

**Table 1:** Study characteristics

Author/s	Title	Publication year	Journal	Country	Setting	Study design	Objectives	Inclusion criteria
DeWitty, Huerta, & Downing	New careers in nursing: Optimizing diversity and student success for the future of nursing	2016	Journal of Professional Nursing	USA	130 nursing schools in 41 states and the District of Columbia	Longitudinal survey	1. What were scholars’ self-rated satisfaction withtheir learning environments?; 2. What were scholars’ self-rated perceptions of the effectiveness of the Pre-Entry Immersion Program (PIP)?; 3. What were scholars’ perceptions of mentoring and leadership development?; 4. What did scholars identify as facilitators and barriers to their academic success?	On the basis of entry dates into their programs and scholarship awards, scholars were assigned to cohorts with similar dates for data collection purposes
Harding, Jamieson, Withington, Hudson, & Dixon	Attracting men to nursing: Is graduate entry an answer?	2018	Nursing Education in Practice	New Zealand	Polytechnic & a University	Qualitative descriptive	Describe the reasons underpinning men’s enrolment in the first three intakes of the first such programin New Zealand	All the men enrolled in the first three cohorts of the graduate entry nursing programme at the authors’ institutions
Jamieson, Harding, Withington, & Hudson	Men entering nursing: Has anything changed?	2019	Nursing Praxis in New Zealand	New Zealand	Polytechnic & a University	Qualitative descriptive	The aim of this study was to describe male nursing students’ understanding of the gender stereotypes associated with nursing	All the men enrolled in the first three cohorts of the graduate entry nursing programme at the authors’ institutions
McKenna & Vanderheide	Graduate entry to practice in nursing: Exploring demographic characteristics of commencing students	2012	Australian Journal of Advanced Nursing	Australia	University	Cross-sectional survey	Examine characteristics of individuals in the first two cohorts undertaking the Master of Nursing Practice at the authors’ university, including demographic details, previous education, and rationale for pursuing change of career, in order to better understand their learning needs	Individuals in the first two cohorts undertaking the Master of Nursing Practice at the authors’ university
Neill, M	Graduate-entry nursing students’ journeys to registered nursing	2012	Nursing Education in Practice	Australia	Australian University	Grounded theory	Examine the experiences of graduate-entry nursingstudents in an Australian university: Stage 1: To describe the decision to pursue nursing; Stage 2: To describe graduate-entry educational experiences; Stage 3: To describe experiences of having practiced as a Registered Nurse	All graduates of a graduate-entry nursing program between the years of 1999 and 2004 now practicing as a registered nurse
Raines, D.	What attracts second degree students to a career in nursing?	2010	The Online Journal of Issues in Nursing	USA	Second degree South-eastern United States	Qualitative descriptive	The purpose of this research study was to explore the self-described factors motivating individuals to seek the opportunity to study nursing in an accelerated, second-degree, nursing program	Members of the first two cohorts of students while they were still prospective students

The objectives and design of each of the six included studies are now described in further detail, highlighting the aspects within each study that particularly inform the motivations to enrol in a GEN programme. DeWitty et al’s [22] longitudinal survey involved the collection of quantitative and qualitative survey data at three time points (*n* = 3335) across 130 schools of nursing in 41 states and the District of Columbia in the USA. These schools of nursing were part of a scholarship program (New Careers in Nursing) in which scholarships were awarded to second-degree accelerated nursing students. In terms of measuring students’ motivations for enrolling in a GEN programme, within the survey, students were asked to rate the benefits of the scholarship on a 5-point Likert scale. In terms of exploring their motivations, students were asked for their reasons for pursuing a nursing degree.

Both New Zealand studies investigated the same cohort of males enrolled in the GEN programmes at the authors’ tertiary education institutions. Harding et al. [18] sought to describe the reasons underpinning men’s enrolment in the first three intakes of the first graduate entry nursing programme in New Zealand, Jamieson et al. [19] extended this work, seeking to describe these male nursing students’ understanding of the gender stereotypes associated with nursing. In terms of questions specifically related to motivation to enrol in a GEN program, for Harding et al. [18], students were asked “Can you tell me about your decision to enrol in fast track graduate entry nursing program at this time”, “Do you think your life stage has influenced your decision to enrol in this course?”, and more broadly, “Can you tell me about your decision to become a nurse?”, “Did you actively seek a ‘non-traditional male career? Can you tell me about this?”, and “At this stage have you given any thought to your potential career as a nurse? For example: What area would you like to work in and why?”. Jamieson et al. [19] extended on Harding et al’s [18] analysis of the questions, “Did you actively seek a ‘non-traditional male career? Can you tell me about this?”

The fourth study, McKenna and Vanderheide [23] examined the characteristics of individuals in the first two cohorts undertaking the Master of Nursing Practice at the authors’ university, including demographic details, previous education, and rationale for pursuing change of career, in order to better understand their learning needs. In terms of investigating motivations, McKenna and Vanderheide [23] explored the reasons surrounding students’ application for entry to the course.

The fifth study, Neill’s [20] grounded theory study examined the experiences of GEN students at an Australian university. There were three stages of the study, the first stage particularly related to the motivations of students enrolling in a GEN programme where the investigator sought “To describe the decision to pursue nursing” with open-ended semi-structured questions such as “What initially attracted you to nursing?”

The final included study, Raines [21], analysed the pre-written stories of prospective GEN programme students seeking admission to a Southeast United States university. Their stories described the factors that influenced their decision to pursue nursing as a second career. Inductive content analysis was used to analyse the stories in relation to the research question, “What are the factors leading to a decision to study nursing?”

### Review findings

Of the six included studies, five reported qualitative data [18–22], and two reported quantitative data [22, 23]. The findings of Harding et al. [18] and Jamieson et al. [19] appeared to report the qualitative analysis of different questions asked of the same study participants, thus both studies were included. Firstly, the qualitative and quantitative findings are reported, then finally the narrative synthesis.

### Qualitative findings

The qualitative findings are presented in Table 2.

**Table 2:** Qualitative findings

Authors	Sample	Demographics	Data collection method	Types of analysis	Qualitative findings about motivation to enrol in Graduate Entry Nursing programme
DeWitty, Huerta, & Downing	3335	Average age 29; 60.8% females; 63.9% had never been married; 71.5% did not have children; most common first degree in physical sciences (28.8%); behavioural sciences (18.1%); health sciences (12.2%); 61.1% did not relocate to enrol in the graduate program.	Survey	Thematic	Open-ended responses to benefits of the New Careers in Nursing scholarship included these five major themes: (a) financial (*n* = 918); (b) lower stress (*n* = 213); (c) goal attainment (*n* = 207); (d) focus on school (*n* = 204); and (e) program opportunities (*n* = 199); many reported reasons for making this transitional career shift, such as: a desire to help others through a nursing career (*n* = 713, 32.8%), fulfilling a long-term desire to become a nurse (*n* = 146, 6.7%), and flexibility of career pathways in nursing (*n* = 362, 16.7%).
Harding, Jamieson, Withington, Hudson, & Dixon	8	Males aged 23–39	Individual semi-structured interviews	Thematic (Braun & Clarke)	Two primary themes: (1) in search of a satisfying career; (2) the time was right
Jamieson, Harding, Withington, & Hudson	8	Males aged 23–39	Semi-structured interviews	Thematic (Braun & Clarke)	The participants were aware of two potent gender scripts with respect to nursing: (1) a dominant stereotype of nursing as women’s work, an associated devaluing of nursing work, and the gender typing of some areas of nursing as being more male appropriate; and (2) the stereotyping of men who are nurses as homosexual. Two further themes were also evident: (3) being disquieted by stereotypes that negatively characterise their career choice; and (4) that of resisting the stereotype, as all the participants spoke of their non-subscription to the script of normative masculinity.
Neill	6	Three females, three males	Electronic interview, open-ended semi-structured questions	Coding / constant data comparison (Strauss & Corbin)	Four categories were identified: long-term interest, caring, desire for change, and change anxiety.
Raines	66	Most were female (86%); 25–40 years (23%); 5–10 years since first Bachelor’s degree (82%); first degree arts and humanities (23%).	Participant written stories	Content	‘What I bring to nursing’, ‘Seeking satisfying work’, and ‘Missing pieces’.

DeWitty et al. [22] identified five themes related to the benefits of receiving a scholarship: (a) financial; (b) lower stress; (c) goal attainment; (d) focus on school; and (e) program opportunities. Their participants reported reasons for making this transitional career shift, such as: a desire to help others through a nursing career, fulfilling a long-term desire to become a nurse, and flexibility of career pathways in nursing. For programme length, one of DeWitty et al’s [22] participants stated “*The most positive aspect of the program was being able to receive a quality education in only 14 months. Being able to get my degree and start my career so soon will mean a much better life for my family and higher personal satisfaction as well*.” [22] (p. S9). For financial benefits, “*Without the scholarship, I would not have been able to finish my first semester in nursing school. It helped me tremendously with paying for tuition, books, and lab fees. Thank you so much!*” [22] (p. S10). For lower stress “*The New Careers in Nursing scholarship allowed me to further my career without the added stress that was very taxing on my family of three. This program is great and ultimately saved me! Without it, I would have struggled more in school.*” [22] (p. S10).

Harding et al. [18] found two primary themes. First, ‘in search of a satisfying career’ with the associated sub-themes: was at a loss; fulfilment through working with and helping people; and a career with options. One participant stated “... *basically just never really had anything you’d call a career.*” [18] (p. 259). The second theme ‘the time was right’ was underpinned by two subthemes: The right time of life; and, the right course. For one participant:

I wanted something that was both practical, but had a political dimension to it, and would ultimately get me back overseas. …

My main long-term objective really relates to the reasons why I got into nursing in the first place, which is I’m interested in taking it back overseas and working in the humanitarian sector [18]. (p.260)

In the subtheme ‘the right course’, participants identified that they were attracted to the shorter timeframe, the academic challenge and that it built upon their previous education, for example, one participant reported “the fact that it was shorter definitely appealed” and he “wanted to get to that quickly … there was definitely a financial element there … and it would help me stand out a bit.” [18] (p.261).

Jamieson et al. [19] found the participants were aware of two potent gender scripts with respect to nursing: (1) a dominant stereotype of nursing as women’s work, an associated devaluing of nursing work, and the gender typing of some areas of nursing as being more male appropriate; and (2) the stereotyping of men who are nurses as homosexual. More specific to motivations, the authors reported two further themes: (3) being disquieted by stereotypes that negatively characterise their career choice; and (4) that of resisting the stereotype, as all the participants spoke of their non-subscription to the script of normative masculinity. For one participant:

I feel deep down I’m a caring person, so it affiliated with my values … When I was tossing it up, I think it was that aspect that appealed to me … yeah, non-traditional and, yeah, it’s a different choice and it is a challenging choice for the social order [19]. (p.24)

Then:

I’m a little bothered by how some people react to it, but personally I’m of the opinion that anyone should be allowed to be what they want. I wanted to be in a role where – I like helping people. This is a career where I can help people. Why shouldn’t I be allowed to do this? Why should this be a problem? [19] (p.25)

Neill [20] identified four categories, three particularly related to motivations: long-term interest, caring, and desire for change. For long-term interest, participants expressed “I had been interested in nursing since childhood” [20] (p.91), and “Nursing has always been in the back of my mind to do one day – had to find the time” [20] (p.91). For caring, participants expressed “I wanted a job caring for people and families” [20] (p.91), and “Seeing what nurses actual [ly] did really fuelled my interest” [20] (p.91). For desire for change, participants expressed “At mid-life I just wanted to care for people in my daily work to find meaning” [20] (p.91), and “My career had absolutely no elements of caring or sense of community contribution” [20] (p.91).

Raines [21] identified ‘What I bring to nursing’, ‘Seeking satisfying work’, and ‘Missing pieces’. For ‘What I bring to nursing’, participants expressed both skills and experiences they had developed in their previous work roles and educational programmes, such as “My communication skills will make me proficient in treating patients and their families” [21] (p.9). For ‘Seeking satisfying work’, participants expressed feeling satisfied and seeing what nurses do as satisfying, such as “I want to feel satisfied at the end of the day, knowing that my work made a difference.” [21] (p.10). For ‘Missing pieces’, participants expressed feelings of responsibility to help others and a desire for more knowledge, such as “Nursing is a career path in which I will be able to pursue my greater responsibility to humanity” and “Becoming a nurse will provide me with knowledge and resources to pursue a deeper level of caring for families” [21] (p.10).

### Quantitative results

The two studies reporting quantitative data were DeWitty et al. [22] and McKenna and Vanderheide [23]. These data are presented in Table 3.

**Table 3:** Quantitative results

Authors	Sample	Demographics	Data collection	Types of analysis	Quantitative findings about motivation to enrol in Graduate Entry Nursing programme
DeWitty, Huerta, & Downing	3335	Average age 29; 60.8% females; 63.9% had never been married; 71.5% did not have children; most common first degree in physical sciences (28.8%); behavioural sciences (18.1%); health sciences (12.2%); 61.1% did not relocate to enrol in the graduate program.	Survey	Descriptive statistics, ANOVA	Scholarships: (1) helped ease students’ financial burden (*n* = 1011); (2) gave them confidence and motivation to succeed in nursing (*n* = 1007); (3) allowed more time devoted to their academic studies (*n* = 1008); and (4) decreased or eliminated hours they needed to work (*n* = 1007). For some, the scholarship served as the deciding factor in their decision to enrol in the nursing program (*n* = 1008).
McKenna & Vanderheide	79	Age 21–25 years (24.1%, *n* = 19); 29.1% (*n* = 23) were male and 69.6% (*n* = 55) female - 42.3% of participants in the first cohort were male.	Survey	Descriptive statistics	1) Main reason for undertaking a nursing course: career stability, diversity of practice and the caring nature of nursing highly; influence of personal experience with the health care system. Availability of family support (32.9%, *n* = 26) and eagerness to become qualified (78.5%, *n* = 62) being the most common influences. 2) Why they chose this course over other available nursing courses (multiple response options): Offered at postgraduate level *(n* = 51, 64.6%), the length of the course was identified by (*n* = 59, 74.9%), the accelerated nature of the course (*n* = 57, 72.2%), location of offering (*n* = 28, 35.4%), because of the university offering it (*n* = 24, 30.4%,) and other reason (*n* = 6, 7.6%).

DeWitty et al’s [22] longitudinal survey found for some students, receipt of a scholarship served as the deciding factor in their decision to enrol in the nursing program. Almost one third of participants reported the scholarships eased their financial burden, and allowed them addtional time to study, because they did not need to work as many hours. Receiving a scholarhsip also gave students confidence and motivation to succeed in nursing. DeWitty et al.’s [22] analysis of the open-ended questions enabled these responses to be further unpacked, as reported in the qualitative findings above. The quantitative results are presented in Table 3.

McKenna and Vanderheide [23] surveyed students (*n* = 79) and found the most common motivator was the availability of family support, and an eagerness to qualify. Other reasons included the length of the course, the accelerated design of the course, the location, and because the university offered it. Six students selected other, but did not elaborate. Just less than half of the participants (46%) had considered nursing as a career for a year or less and 39% for 2 years or longer.

### Narrative synthesis of qualitative and quantitative data

The thematic analysis identified four themes: finding meaning and purpose through altruism and caring; seeking a satisfying career; looking for a change in direction; reduced financial burden.

#### Finding meaning and purpose through altruism and caring

A common motivator for individuals attracted to GEN programmes was the desire to help others [20–22], at both at an individual and societal level [21]. Participants in Harding’s et al. [18] study recognised this as gaining a sense of fulfillment through helping people. One participant explained “I was actually being helpful - I was doing something worth doing” [18] (p.259). Others likened this to gaining a sense of satisfaction from their career recognising that this had been missing from their previous roles [18]. Jamieson et al. [19] found participants in their study, who were all male, wanted to be in a caring role as they saw themselves as caring people. Another participant questioned why they shouldn’t, stating; “I like helping people, this is a career where I can help people. Why shouldn’t I be allowed to do this?” [19] (p.25), with a third participant recognising that “caring behaviours are innate to both genders” [19] (p.25). Students reflected on the skills and knowledge they bought from their previous employment and study, suggesting their enrolment involved significant self-reflection [21].

#### Seeking a satisfying career

Some participants had previously sought caregiving or support work which helped them to recognise their enjoyment of satisfying work [18]. Participants in Raines [21] study explained that satisfaction (or lack of it) was related to their current work activities and their personal observation that nurses work was satisfying. Participants had observed nurses work in their previous employment and volunteer roles [21].

Another important influence for participants was meeting their goals and interests [20, 22]. While only nominally reported on in DeWitty’s et al. [22] study 6.7% of the participants nominated a long-held desire to be a nurse as a motivation for undertaking the programme. Often these ambitions had been present for several years with participants expressing they had wanted to do nursing for a long time [19], some since childhood [18, 20].

The flexibility of future career options was attractive to participants [18, 22, 23]. In particular, participants (65.8%) nominated career stability as a key reason for enrolment [23]. Participants also recognised that nursing offers the opportunities to move both vertically and laterally. One participant commented, “the very thing about nursing is that if you’re dissatisfied with something you can change” [18] (p.260).

#### Looking for a change in direction

This theme reflected participants recognition that not only did they want a career change [20], but that it was also the right time in their lives to do the course [18]. Participants decided the course was ‘right’ for several reasons; the shorter two-year course was financially attractive, it was a ‘faster route’ to finish, and the postgraduate level was appealing, with participants feeling the advanced level might enhance their career prospects [18] (p.261).

Participants current employment situation influenced their decision to change direction due to dissatisfaction with their current employment [21, 23] or with their career [18], while some participants enrolled because they were currently unemployed [23]. However, while wanting to change career direction, participants in Neill’s [20] study expressed some hesitancy regarding the impact of the change on their family and financial security.

#### Reduced financial burden

**Fig. 2:**
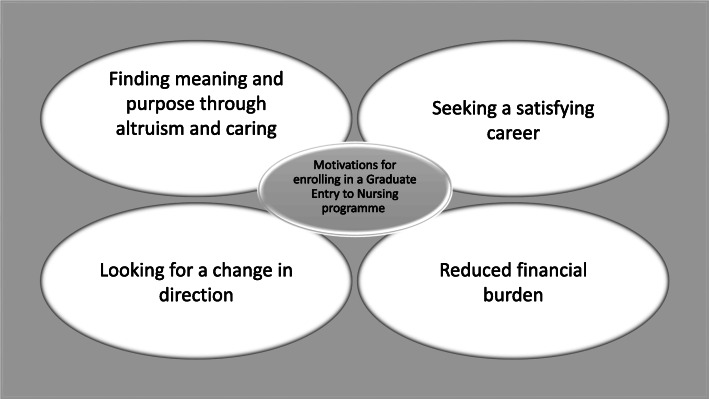
Motivations for enrolling in a Graduate Entry to Nursing programme

The two-year accelerated course was reported as financially appealing [18]. Additionally, scholarships were seen to ease the financial burden, give confidence and motivation to succeed in nursing, allowed more time devoted to academic studies, and decreased or eliminated hours needed to work [22]. Figure 2 displays the four themes that were identified.

## Discussion

The papers included in this review were primarily from journals well-known to nursing. A limitation was that none of the included studies specifically asked study participants’ their motivations for enrolling in a GEN programme. More commonly the participants’ motivations were reported when responding to questions about closely related areas such as their decision to pursue nursing as a career. Furthermore, the quality appraisal highlighted a range of study strengths, but also limitations such as lack of 1) philosophical perspectives, 2) clarity in research methodology, and 3) positioning of the researcher culturally, theoretically, and within the research. Additionally, there may have been considerable bias in the results of Raine’s [21] retrospective document review of the prospective students’ stories regardless of their control measures. Despite these limitations, the qualitative and quantitative data provided several new insights into motivators for enrolling in a GEN programme. Four themes of motivation were found, finding meaning and purpose through altruism and caring, seeking a satisfying career, looking for a change in direction and reduced financial burden.

The first theme, finding meaning and purpose through altruism and caring, was evident in the research of all six of the studies [18–23]. This was expressed by participants in a range of ways such as helping others, caring, and seeking fulfilment. Motivated by caring is often reported by students entering a career as a nurse in undergraduate programmes [24], and seemingly is also evident in those entering GEN programmes. Finding meaning in life is proposed to involve multiple stages of making sense of experiences, then integrating experiences to develop an understanding of ourselves, then determining how we fit into the world around us [25]. From finding meaning stems purpose, the motivation to actively pursue goals reflecting one’s identity [25]. The New Zealand studies focusing on males entering GEN programmes particularly resonate with this, notably, the themes of ‘males resisting the stereotype’ [19], and ‘fulfilment through working with and helping people’ [18].

The second and third themes focused on seeking a satisfying career or a change in career direction. Those seeking a satisfying career [18, 21–23] expressed enduring interest in meeting long-term goals and seeking satisfying work that has flexibility and options. Those looking for a change in direction [18, 20, 21, 23] expressed current job dissatisfaction, unemployment, feeling at a loss, and missing pieces. Work has implications for both health and wellbeing, as a means of survival, relatedness, and self-determination [26]. When job characteristics align with employee needs and abilities, good person-job fit is more likely [27].

Intrinsic and extrinsic motivations for enrolment in nursing programmes have been frequently reported for those entering undergraduate programmes [28, 29]. Most commonly, intrinsic factors include the desire to help others, job opportunities, and their experience of caring for others [28–30]. Extrinsic factors include job security [29], and earnings [29, 30]. Most participants in the included studies were above the age of 21 (where reported), and of these many were over the age of 25. This age reflects the common age of students in GEN programmes [31, 32], a consequence of the time taken to obtain their first degree and then those that elect to enrol after a previous career. More broadly, students aged 21 years or less and those aged 28 years or more reported different motivations for choosing a nursing career, whereby older students reported higher levels of intrinsic motivation and identified a deliberate decision-making process [33]. These higher levels of intrinsic motivation were evident in this review’s findings, where participants shared statements of very deliberate long-term goal setting.

The final theme of reduced financial burden in this review was related to the reduced time in the accelerated pathway [18], and the potential benefits of one scholarship offered in USA [22]. Barriers to enrolling were loosely mentioned in the review studies and invariably these pertained to finances [20]. Incentives to address the shortage of nursing graduates are not new, for example, in Australia the federal government offer Commonwealth Supported Places which provides funding to support students’ places in some nursing programmes [34]. It is possible that those people loosely motivated to enroll may find scholarships and other forms of fee assistance support them to take that next career step towards nursing.

Notably, there were very few studies identified in this review that investigated the motivations to enrol in a GEN programme, and those few studies were from only three of those countries that actually offer accelerated programmes. In the New Zealand context this paucity of research likely reflects the recent implementation of GEN programmes in 2014 [35]. In fact, the only New Zealand study investigated the reasons men enrolled in the GEN programmes. Jamieson et al. [35] have replicated McKenna and Vanderheide’s [23] study and this is currently in press. This study reports the demographics and characteristics of five cohorts from one programme in New Zealand from 2014 to 2018. The main contribution of this recent research in terms of the motivation of students to enrol in a GEN programme were to reinforce the finding that a key motivation was wanting to work in a diverse and caring profession. The authors highlight the alignment of their findings with other research in this population [35].

The factors that motivate GEN students to commence their programme may be both individualised and context-dependent, suggesting that developing an understanding of these multi-dimensional motivators is complex. Exploring the motivators and then longitudinally investigating how these motivators are perceived by the learners to be enablers and barriers to their learning experience is an important next research step in developing strategies for both recruitment and retention of the GEN students.

### Limitations

The review title was not registered with The Joanna Briggs Institute due to their limitations on the types of bodies that are able to register reviews. The protocol was not registered with PROSPERO due to their limitations to systematic review registration only. Given the potential influence of the researchers on the exploration, analysis and interpretation of the data, two independent reviewers were used at significant stages of the narrative synthesis (analysis, interpretation) and a sample of the analysis is provided as supplementary materials (see Supplementary File 3) to enable readers to make their own judgements regarding decisions made during the research process.

## Conclusions

Internationally there is a dearth of evidence regarding the motivations to enrol in a GEN programme. This scoping review has clearly described existing evidence about the factors that motivate GEN candidates to enrol. Of those few studies identified, GEN candidates were found to be motivated by finding meaning and purpose through altruism and caring, seeking a satisfying career, looking for a change in direction and the reduced financial burden of a shortened two-year degree. These preliminary insights into the decision-making surrounding starting a GEN programme highlight the influence of their experiences and values. There remains a need for further specific and focused research to deepen our understanding of the factors motivating students to enrol in GEN programmes. This knowledge will enable education providers to target programme recruitment strategies, ultimately contributing to the development of the future nursing workforce, thus establishing GEN programmes as a sustainable approach to addressing the global workforce shortage.

## Supplementary Information

**Additional file 1: Supplementary file 1.** Search used in SCOPUS. **Supplementary file 2.** Table 1. Critical appraisal judgements for the cross-sectional survey. Questions from JBI critical appraisal tool: Q1) Were the criteria for inclusion in the sample clearly defined?; Q2) Were the study subjects and the setting described in detail?; Q3) Was the exposure measured in a valid and reliable way?; Q4) Were objective, standard criteria used for measurement of the condition?; Q5) Were confounding factors identified?; Q6) Were strategies to deal with confounding factors stated?; Q7) Were the outcomes measured in a valid and reliable way?; Q8) Was appropriate statistical analysis used?. Table 2 Critical appraisal judgements for the longitudinal study. Questions from JBI critical appraisal tool: Q1) Was the sample frame appropriate to address the target population?; Q2) Were study participants sampled in an appropriate way?; Q3) Was the sample size adequate?; Q4) Were the study subjects and the setting described in detail?; Q5) Was the data analysis conducted with sufficient coverage of the identified sample?; Q6) Were valid methods used for the identification of the condition?; Q7) Was the condition measured in a standard, reliable way for all participants?; Q8) Was there appropriate statistical analysis?; Q9) Was the response rate adequate, and if not, was the low response rate managed appropriately?. Table 3 Critical appraisal judgements for the qualitative studies. Questions from JBI critical appraisal tool: Q1) Is there congruity between the stated philosophical perspective and the research methodology? Q2) Is there congruity between the research methodology and the research question or objectives?; Q3) Is there congruity between the research methodology and the methods used to collect data?; Q4) Is there congruity between the research methodology and the representation and analysis of data?; Q5) Is there congruity between the research methodology and the interpretation of results?; Q6) Is there a statement locating the researcher culturally or theoretically?; Q7) Is the influence of the researcher on the research, and vice- versa, addressed?; Q8) Are participants, and their voices, adequately represented?; Q9) Is the research ethical according to current criteria or, for recent studies, and is there evidence of ethical approval by an appropriate body?; Q10) Do the conclusions drawn in the research report flow from the analysis, or interpretation, of the data?. **Supplementary file 3.** Table of themes (R1).

## Data Availability

Not applicable as there was no primary data collected.

## Abbreviations

CINAHL: The Cumulative Index of Nursing and Allied Health Literature, a bibliographic database
ERIC: Education Resources Information Center
GEN: Graduate Entry Nursing
MEDLINE: Bibliographic database for life sciences and biomedical information
PRISMA: Preferred Reporting Items for Systematic Reviews and Meta-Analysis Protocol
SCOPUS: Scopus is an abstract and citation database of peer-reviewed literature
USA: United States of America
UK: United Kingdom
