# Supplementary material for: What motivates people to commence a graduate entry nursing programme: a mixed method scoping review
Source: BMC Nurs. 2021 Mar 20;20:47. doi: 10.1186/s12912-021-00564-9 (PMC7981909; doi:10.1186/s12912-021-00564-9)
Supplement: Supplementary file 1 — Additional file 1: Supplementary file 1. Search used in SCOPUS. Supplementary file 2. Table 1. Critical appraisal judgements for the cross-sectional survey. Questions from JBI critical appraisal tool: Q1) Were the criteria for inclusion in the sample clearly defined?; Q2) Were the study subjects and the setting described in detail?; Q3) Was the exposure measured in a valid and reliable way?; Q4) Were objective, standard criteria used for measurement of the condition?; Q5) Were confounding factors identified?; Q6) Were strategies to deal with confounding factors stated?; Q7) Were the outcomes measured in a valid and reliable way?; Q8) Was appropriate statistical analysis used?. Table 2 Critical appraisal judgements for the longitudinal study. Questions from JBI critical appraisal tool: Q1) Was the sample frame appropriate to address the target population?; Q2) Were study participants sampled in an appropriate way?; Q3) Was the sample size adequate?; Q4) Were the study subjects and the setting described in detail?; Q5) Was the data analysis conducted with sufficient coverage of the identified sample?; Q6) Were valid methods used for the identification of the condition?; Q7) Was the condition measured in a standard, reliable way for all participants?; Q8) Was there appropriate statistical analysis?; Q9) Was the response rate adequate, and if not, was the low response rate managed appropriately?. Table 3 Critical appraisal judgements for the qualitative studies. Questions from JBI critical appraisal tool: Q1) Is there congruity between the stated philosophical perspective and the research methodology? Q2) Is there congruity between the research methodology and the research question or objectives?; Q3) Is there congruity between the research methodology and the methods used to collect data?; Q4) Is there congruity between the research methodology and the representation and analysis of data?; Q5) Is there congruity between the research methodology and the interpretation o [file 12912_2021_564_MOESM1_ESM.docx]

Supplementary file 1

**Search used in SCOPUS**

| **Terms used** | **Hits** |
| --- | --- |
| ( ( ( TITLE-ABS-KEY ( ( masters ) W/3 ( program* OR nurs* ) ) ) OR ( TITLE-ABS-KEY ( "graduate entry" OR "direct entry" OR "G msn" OR "MSN entry" OR "MNSc" OR "second degree" OR "accelerated master*" ) ) ) AND ( TITLE-ABS-KEY ( nursing OR nurse OR nurses ) ) ) AND ( TITLE-ABS-KEY ( motivat* OR enabl* OR barrier* OR facilitat* OR perception* OR perceive* OR aspiration* OR attitude* ) ) ) AND ( TITLE-ABS-KEY ( "enrol*" OR "becom*" OR "begin*" OR "commenc*" OR "start*" OR "enter*" OR "entry" ) ) AND ( LIMIT-TO ( EXACTKEYWORD, "Human" ) OR LIMIT-TO ( EXACTKEYWORD, "Humans" ) ) | 199 |

Supplementary file 2.

Table 1. Critical appraisal judgements for the cross-sectional survey

| Study | Reviewer | Q1 | Comments | Q2 | Comments | Q3 | Comments | Q4 | Comments | Q5 | Comments | Q6 | Comments | Q7 | Comments | Q8 | Comments |
| --- | --- | --- | --- | --- | --- | --- | --- | --- | --- | --- | --- | --- | --- | --- | --- | --- | --- |
| McKenna 2012 | R2 | Yes | No exclusion criteria reported | Yes |  | Yes | N/A for design | Yes | N/A for design of study | Yes | N/A for study design | Yes | N/A for study design | No | Validity and reliability of measures not reported, e.g., job satisfaction | No | Lacked reporting of variation, e.g., standard deviation |
| McKenna 2012 | R1 | Yes | No exclusion criteria reported | Yes | Study participants were described demographically and by gender | Yes | n/a | Yes | n/a | Yes | N/A for this study as a survey | Yes | N/A | No | No previously validated tool was used to measure results eg job satisfaction | No | Lack of reporting such as the mean and standard deviation. |
| McKenna 2012 | Consensus | Yes | No exclusion criteria reported | Yes | Study participants were described demographically and by gender | Yes | N/A for design | Yes | N/A for design of study | Yes | N/A for study design | Yes | N/A for study design | No | No previously validated tool was used to measure results eg job satisfaction | No | Lack of reporting such as the mean and standard deviation. |

Questions from JBI critical appraisal tool: Q1) Were the criteria for inclusion in the sample clearly defined?; Q2) Were the study subjects and the setting described in detail?; Q3) Was the exposure measured in a valid and reliable way?; Q4) Were objective, standard criteria used for measurement of the condition?; Q5) Were confounding factors identified?; Q6) Were strategies to deal with confounding factors stated?; Q7) Were the outcomes measured in a valid and reliable way?; Q8) Was appropriate statistical analysis used?

Table 2. Critical appraisal judgements for the longitudinal study

| Study | Reviewer | Q1 | Comments | Q2 | Comments | Q3 | Comments | Q4 | Comments | Q5 | Comments | Q6 | Comments | Q7 | Comments | Q8 | Comments | Q9 | Comments |
| --- | --- | --- | --- | --- | --- | --- | --- | --- | --- | --- | --- | --- | --- | --- | --- | --- | --- | --- | --- |
| DeWitty 2016 | R2 | Yes |  | Yes |  | unclear | Total population not reported | Yes |  | No | Some sub-groups had very low response rates | Yes | N/A for study design | Yes | N/A for study design | Yes | Means and standard deviations are reported | No | No discussion of dropouts |
| DeWitty 2016 | R1 | Yes |  | Yes |  | Yes |  | Yes |  | Yes |  | Yes |  | Yes |  | Yes |  | Yes |  |
| DeWitty 2016 | Consensus | Yes |  | Yes |  | unclear | Total population not reported | Yes |  | No | Some sub-groups had very low participation rates | Yes | N/A for study design | Yes | N/A for study design | Yes | Means and standard deviations are reported | No | No discussion of dropouts |

Questions from JBI critical appraisal tool: Q1) Was the sample frame appropriate to address the target population?; Q2) Were study participants sampled in an appropriate way?; Q3) Was the sample size adequate?; Q4) Were the study subjects and the setting described in detail?; Q5) Was the data analysis conducted with sufficient coverage of the identified sample?; Q6) Were valid methods used for the identification of the condition?; Q7) Was the condition measured in a standard, reliable way for all participants?; Q8) Was there appropriate statistical analysis?; Q9) Was the response rate adequate, and if not, was the low response rate managed appropriately?

Table 3. Critical appraisal judgements for the qualitative studies

| Study | Reviewer | Q1 | Comments | Q2 | Comments | Q3 | Comments | Q4 | Comments | Q5 | Comments | Q6 | Comments | Q7 | Comments | Q8 | Comments | Q9 | Comments | Q10 |
| --- | --- | --- | --- | --- | --- | --- | --- | --- | --- | --- | --- | --- | --- | --- | --- | --- | --- | --- | --- | --- |
| Raines 2011 | R2 | No | No philosophical perspective nor research methodology stated | No | No research methodology stated | No | No research methodology stated | No | No research methodology stated | No | No research methodology stated | No | Researcher not located | Yes |  | Yes | In tabular form and aligned with analysis | Yes | Institutional Review Board | Yes |
| Raines 2011 | R1 | No | Not stated | No |  | No |  | No |  | No |  | No | not mentioned | Yes | The researcher asked colleagues to validate the analysis | Yes |  | Yes |  | Yes |
| Raines 2011 | Consensus | No | No philosophical perspective nor research methodology stated | No | No research methodology stated | No | No research methodology stated | No | No research methodology stated | No | No research methodology stated | No | Researcher not located | Yes | The researcher asked colleagues to validate the analysis | Yes | In tabular form and aligned with analysis | Yes | Institutional Review Board | Yes |
| Neill 2012 | R2 | Yes |  | Yes |  | Yes |  | Yes |  | Yes |  | No | Nil statement | No | No statement | Yes |  | Yes |  | Yes |
| Neill 2012 | R1 | Yes |  | Yes | Congruity as the study was interested in experience | Yes | Used semi-structured questions | Yes |  | Yes | Constant data comparison was used. | No | The researcher did not locate himself | No |  | Yes |  | Yes |  | Yes |
| Neill 2012 | Consensus | Yes |  | Yes |  | Yes |  | Yes |  | Yes |  | No | The researcher did not locate himself | No | No statement | Yes |  | Yes |  | Yes |
| Harding 2018 | R2 | No |  | No |  | No |  | No |  | No |  | No | Not stated | No |  | Yes |  | Yes |  | Yes |
| Harding 2018 | R1 | No | No philosophical perspective nor research methodology stated | No | No research methodology stated | No | No research methodology stated | No | No research methodology stated | No | No research methodology stated | No | Not located | No | Not addressed | Yes |  | Yes |  | Yes |
| Harding 2018 | Consensus | No | No philosophical perspective nor research methodology stated | No | No research methodology stated | No | No research methodology stated | No | No research methodology stated | No | No research methodology stated | No | Not stated | No | Not addressed | Yes |  | Yes |  | Yes |
| Jamieson 2019 | R2 | No | No philosophical perspective nor research methodology stated | No | No research methodology stated | No | No research methodology stated | No | No research methodology stated | No | No research methodology stated | No | Researcher not located | No | Influence not addressed | Yes |  | Yes |  | Yes |
| Jamieson 2019 | R1 | No |  | No |  | No |  | No |  | No |  | No |  | No |  | Yes |  | Yes |  | Yes |
| Jamieson 2019 | Consensus | No | No philosophical perspective nor research methodology stated | No | No research methodology stated | No | No research methodology stated | No | No research methodology stated | No | No research methodology stated | No | Researcher not located | No | Influence not addressed | Yes |  | Yes |  | Yes |

Questions from JBI critical appraisal tool: Q1) Is there congruity between the stated philosophical perspective and the research methodology?Q2) Is there congruity between the research methodology and the research question or objectives?; Q3) Is there congruity between the research methodology and the methods used to collect data?; Q4) Is there congruity between the research methodology and the representation and analysis of data?; Q5) Is there congruity between the research methodology and the interpretation of results?; Q6) Is there a statement locating the researcher culturally or theoretically?; Q7) Is the influence of the researcher on the research, and vice- versa, addressed?; Q8) Are participants, and their voices, adequately represented?; Q9) Is the research ethical according to current criteria or, for recent studies, and is there evidence of ethical approval by an appropriate body?; Q10) Do the conclusions drawn in the research report flow from the analysis, or interpretation, of the data?

Supplementary file 3

| Number | Themes (R2) | Authors themes |
| --- | --- | --- |
| 1 | Finding meaning and purpose through altruism and caring: “help others” (DeWitty et al); Caring (Neill); ‘What I bring to nursing’ (Raines) | “help others” (DeWitty et); Caring (Neill); ‘What I bring to nursing’ (Raines) |
| 2 | The timing was right to address long term goals: meeting long term goals (DeWitty); Good timing: The right time of life (Harding); Long-term interest (Neill) | Good timing: The right time of life (Harding); Long-term interest (Neill) |
| 3 | There was support to ease the financial burden: “Scholarship: ease their financial burden; gave them confidence and motivation to succeed in nursing; allowed more time devoted to their academic studies; and decreased or eliminated hours they needed to work (DeWitty) | “Scholarship: ease their financial burden; gave them confidence and motivation to succeed in nursing; allowed more time devoted to their academic studies; and decreased or eliminated hours they needed to work (DeWitty) |
| 4 | Seeking a satisfying career or career change: ‘was at a loss’; ‘fulfilment through working with and helping people’; and ‘a career with options’ (Harding); ‘Seeking satisfying work’ (Raines); Desire for change (Neill); ‘Missing pieces’ (Raines); The course: Right course; and, the right course (Harding); Change anxiety (Neill); flexibility of career pathways (DeWitty) | ‘was at a loss’; ‘fulfilment through working with and helping people’; and ‘a career with options’ (Harding); ‘Seeking satisfying work’ (Raines); Desire for change (Neill); ‘Missing pieces’ (Raines); The course: Right course; and, the right course (Harding); Change anxiety (Neill); flexibility of career pathways (DeWitty) |
|  |  |  |
| 5 | Resisting the stereotypes: For males in the New Zealand context, two themes included: males being disquieted by stereotypes that negatively characterise their career choice (Jamieson) and males resisting the stereotype - non-subscription to the script of normative masculinity (Jamieson) | For males in the New Zealand context, two themes included: males being disquieted by stereotypes that negatively characterise their career choice (Jamieson) and males resisting the stereotype - non-subscription to the script of normative masculinity (Jamieson) |

Table of themes (R1)

| **Themes/Authors** | **Harding et al. (2018)** | **Jamieson et al. (2019)** | **Neill, (2012)** | **Raines (2010)** | **DeWitty et al. (2016)** |
| --- | --- | --- | --- | --- | --- |
| **Care for others/help** | Fulfilment through working with others | I'm a caring person | Wanted a caring job | Help other focus on a whole person | Help others |
| **Career; long-term interest** | In search of a satisfying career | Thought about it when he was younger | Attracted to it as a career over time | Seeking a satisfying career | Long-term desire |
| **Desire for change/ time was right** | Time was right |  | Needing a change | Desire for more knowledge |  |
| **Previous employment** | Background in voluntary work |  |  |  |  |
